# Prebiotics and probiotics – the importance of branding

**DOI:** 10.3402/mehd.v23i0.18566

**Published:** 2012-06-18

**Authors:** Ross Crittenden

**Affiliations:** Valio Ltd, Helsinki, Finland

## Abstract

The costs of developing a probiotic or prebiotic ingredient have always been substantial. Ingredient characterization, evaluation of technological and physiological properties, and demonstrations of safety and clinical efficacy require expensive research. The demanding regulatory requirements imposed by EFSA raise the bar even higher so that the costs of acquiring the necessary clinical evidence to support labeling of these food ingredients is approaching that of pharmaceuticals. In order to justify investment in such expensive clinical development, companies require certainty that they can gain a return on investment. Patenting can provide some protection but is not always possible to patent ingredients, and the period of protection is limited. All ingredients eventually face the prospect of commoditization once patents expire. Branding strategies offer one means of maintaining adequate product differentiation to protect market share and margins over the long term.

